# Adverse weather conditions and fatal motor vehicle crashes in the United States, 1994-2012

**DOI:** 10.1186/s12940-016-0189-x

**Published:** 2016-11-08

**Authors:** Shubhayu Saha, Paul Schramm, Amanda Nolan, Jeremy Hess

**Affiliations:** 1 Climate and Health Program, Division of Environmental Hazards and Health Effects, National Center for Environmental Health, Centers for Disease Control and Prevention, 4770 Buford Hwy, MS F59, Atlanta, GA 30341 USA; 2 College of Public Health, Rhodes Hall, Health Sciences Campus, University of Georgia, Athens, GA 30602 USA; 3 Department of Global Health, Division of Emergency Medicine, Department of Environmental and Occupational Health Sciences, University of Washington, 4225 Roosevelt Way NE #100, Seattle, WA 98105 USA

**Keywords:** Weather, Fatal crash, Injury, Precipitation

## Abstract

**Background:**

Motor vehicle crashes are a leading cause of injury mortality. Adverse weather and road conditions have the potential to affect the likelihood of motor vehicle fatalities through several pathways. However, there remains a dearth of assessments associating adverse weather conditions to fatal crashes in the United States. We assessed trends in motor vehicle fatalities associated with adverse weather and present spatial variation in fatality rates by state.

**Methods:**

We analyzed the Fatality Analysis Reporting System (FARS) datasets from 1994 to 2012 produced by the National Highway Traffic Safety Administration (NHTSA) that contains reported weather information for each fatal crash. For each year, we estimated the fatal crashes that were associated with adverse weather conditions. We stratified these fatalities by months to examine seasonal patterns. We calculated state-specific rates using annual vehicle miles traveled data for all fatalities and for those related to adverse weather to examine spatial variations in fatality rates. To investigate the role of adverse weather as an independent risk factor for fatal crashes, we calculated odds ratios for known risk factors (e.g., alcohol and drug use, no restraint use, poor driving records, poor light conditions, highway driving) to be reported along with adverse weather.

**Results:**

Total and adverse weather-related fatalities decreased over 1994–2012. Adverse weather-related fatalities constituted about 16 % of total fatalities on average over the study period. On average, 65 % of adverse weather-related fatalities happened between November and April, with rain/wet conditions more frequently reported than snow/icy conditions. The spatial distribution of fatalities associated with adverse weather by state was different than the distribution of total fatalities. Involvement of alcohol or drugs, no restraint use, and speeding were less likely to co-occur with fatalities during adverse weather conditions.

**Conclusions:**

While adverse weather is reported for a large number of motor vehicle fatalities for the US, the type of adverse weather and the rate of associated fatality vary geographically. These fatalities may be addressed and potentially prevented by modifying speed limits during inclement weather, improving road surfacing, ice and snow removal, and providing transit alternatives, but the impact of potential interventions requires further research.

**Electronic supplementary material:**

The online version of this article (doi:10.1186/s12940-016-0189-x) contains supplementary material, which is available to authorized users.

## Background

Motor vehicle fatalities were the leading cause of unintentional injury deaths in the United States when data across all age groups was combined from 1999 to 2012 [1]. Centers for Disease Control and Prevention (CDC) calculated the medical spending and productivity loss associated with fatal and non-fatal motor vehicle injuries to exceed 99 billion in 2005 [2]. Given this disease burden and cost, the CDC have singled out reducing motor vehicle injuries as a high priority “winnable battle” for public health [3].

Weather variability proved to be an important factor when the relationship between traffic volume and road traffic injuries were analyzed in Southern California [4]. William Haddon pioneered injury epidemiology with a systematic classification of etiology into phases (pre-event, event, and post-event) and factors (host, agent, and environment) affecting road traffic injuries to facilitate prevention planning [5, 6]. However, isolating and managing risks related to environmental factors, particularly weather, has been challenging. It can be difficult to determine exposure to weather with precision [7], compounded by the potential correlation between weather and other factors, like road utilization, driver behavior, road conditions, and post-event factors such as delayed response time for emergency medical services [8, 9].

The Federal Highway Administration defines “weather-related” crashes as those that occur in adverse weather (e.g., rain, snow, sleet, fog, or a combination) or slick road conditions (e.g., wet, snowy, slushy, or icy) [10]. Such conditions can affect all phases of road traffic injury occurrence via multiple impacts on the host (e.g., decreased visibility), the agent (e.g., increased stopping distance), and the environment (icy or wet road surfaces) [11].

The association between adverse weather and motor vehicle fatalities is not clearly defined as studies indicate both increase [12–14] and a decrease [15, 16] in crash likelihood and/or severity during certain adverse weather conditions. There is limited availability of national estimates of motor vehicle fatalities attributable to adverse weather. One report estimates that 24 % of all crashes in the United States from 1995 to 2005 were weather-related, with an associated annual average of 7,400 deaths and 673,000 injuries [17]. Pisano et al. (2003) reported that the combination of adverse weather and poor pavement conditions contributed to 18 % of fatal crashes and 22 % of injury crashes annually in the United States [11].

This study is novel as it analyzes weather-related motor vehicle fatality information for 19 consecutive years (1994–2012) for the entire continental United States, compares fatalities associated with different types of adverse weather (e.g., snow/ice and rain) and no adverse weather condition, and examines spatial and temporal variation in the rates of such fatalities. The study uses established epidemiologic methods to identify the potential contribution of adverse weather to such fatalities. The state-specific information on fatality rates could help state health departments identify the nature and extent of the injury burden in their jurisdictions and institute appropriate interventions.

## Methods

We used the Fatality Analysis Reporting System (FARS) datasets for 1994–2012 from the National Highway Traffic Safety Administration (NHTSA) [18]. FARS contains data on all traffic crashes on public roads (including motor cycles, passenger vehicles, trucks, and pedestrians) in the United States which resulted in at least one death within 30 days of the crash. The data record contains personal and behavioral information for individuals involved in the crash, and contextual variables indicating the physical factors at the crash location and environmental factors at the time of the crash. We downloaded datasets for each year from the NHTSA website, and used SAS 9.2 (Cary, NC) to compile and analyze the data.

### Identification of adverse weather conditions

For every crash, FARS contains information collected from police reports on reported environmental and road conditions. These conditions are not subsequently cross-referenced with observed meteorological data. Each record indicates an environmental condition around the time of the crash: clear, cloudy, snow, rain, sleet, or fog. For road conditions, each record indicates dry, wet, snow/slush, ice, or sand/dirt/oil. The adverse road conditions of wet, snow/slush, and ice were defined to have an environmental precursor. The specific criteria used to indicate these conditions changed in FARS across the study years, and we modified our criteria to identify adverse environmental and road conditions accordingly (see Additional file 1 for details on variable definitions obtained from FARS data manual http://www-nrd.nhtsa.dot.gov/Pubs/811855.pdf). We focused on four specific weather-related conditions-rain and snow for adverse environmental conditions (thus covering 86 % of all crashes with adverse environmental conditions), and wet and icy for adverse road conditions (thus covering 98 % of all crashes with adverse road conditions with the rest being ‘unspecified conditions’ mostly). Unless explicitly stated, we use the term “adverse weather” to characterize crashes when at least one of these four conditions was reported. We also combined (i) rain and wet road conditions and (ii) snow and icy road conditions for sub-analyses since the prevalence of these conditions would vary geographically and seasonally.

We counted the number of fatalities involved in all crashes as well as those with a reported adverse weather condition for the years 1994–2012. Using information on annual vehicle miles traveled (VMT) for the US [19] for these years, we estimated the annual rates of total fatalities and those associated with adverse weather conditions per billion VMT. We then examined the distribution of these fatalities by months to identify seasonal patterns.

Among the multiple risk factors that are associated with the likelihood of fatal crashes, we want to examine the importance of adverse weather as a risk factor. If other risk factors were systematically correlated with adverse weather conditions at the time of fatal crashes, then the potential association between adverse weather and fatalities would be tenuous. For each crash, we extracted information on the age and gender of individuals who died. We identified if involvement of alcohol, drugs, no use of restraint, speeding or prior driving infringements were reported for each of the fatal crashes. Based on the location and time of each crash, we identified if the crash happened on a highway or under poor light conditions (see Additional file 1 for details on variable definitions obtained from FARS data manual http://www-nrd.nhtsa.dot.gov/Pubs/811855.pdf). Male drivers and the other risk factors mentioned above were dichotomized to indicate their presence or absence. We calculated the Mantel-Haenszel odds ratio (OR) for each risk factor being reported with adverse weather conditions in the event of a fatal crash. This paired comparison helps identify if any of the known risk factors for fatal crashes are correlated with adverse weather, thus confounding the potential association between adverse weather and fatal crashes. An OR of less than one would indicate a lower likelihood of the risk factor being reported for crashes during adverse weather compared to non-adverse weather conditions. We estimated these ORs for all weather conditions, as well as for rain/wet road and snow/icy road conditions separately. Due to concerns of multicollinearity between the dichotomized risk factors, we avoided using logistic regressions with these risk factors as the explanatory variables.

In order to examine the spatial variation in adverse weather related fatalities, we created state-specific (i) total fatality rates and (ii) fatality rates associated with adverse weather per 10,000 vehicle miles travelled (VMT) for 1994–2011 [19]. We used annual VMT estimates for each state that was available from 1994 to 2011. Unfortunately, the VMT estimates were not available separately for adverse weather. We created state-level maps for these two fatality rates using ARCGIS 9.2 (Redlands, CA). We then estimated the rank correlation between the two rates to assess if the spatial patterns in fatality rates associated with adverse weather were different than the overall fatality rates.

## Results

We observed an overall decrease in motor vehicle fatalities from 40,716 in 1994 to 33,561 in 2012 (Table 1). Even though total vehicle miles traveled increased over the study period, the rate of total motor vehicle fatalities per billion VMT decreased from 17.3 in 1994 to 11.3 in 2012. A similar pattern was found for fatalities associated with adverse weather, as the number of fatalities decreased from 7,152 in 1994 to 4,401 in 2012. The rates of fatalities associated with adverse weather per billion VMT decreased from 3.0 in 1994 to 1.5 in 2012. The proportion of total fatalities that were associated with adverse weather decreased from 17.6 % in 1994 to 13.1 % in 2012 with an overall downward trend over the study years. On average, rain/wet conditions were reported for 78 % of fatalities associated with adverse weather, the rest being icy/snow conditions.

**Table 1:** Number of total fatalities with reported adverse weather conditions (FARS: 1994-2012)

Year	Vehicle miles traveled (billion miles)	Total fatality	Rate of total fatality per billion VMT^a^	Fatalities associated with adverse weather conditions		Rate of fatality associated with adverse weather per billion VMT^a^	Proportion of fatalities associated with adverse weather
				Rain or Wet	Snow or Icy		
1994	2358	40716	17.3	5590	1562	3.0	17.6
1995	2423	41817	17.3	5648	1760	3.1	17.7
1996	2482	42065	16.9	5712	2006	3.1	18.3
1997	2560	42013	16.4	5992	1804	3.0	18.6
1998	2625	41501	15.8	5719	1198	2.6	16.7
1999	2679	41717	15.6	4924	1246	2.3	14.8
2000	2747	41945	15.3	5014	1718	2.5	16.0
2001	2796	42196	15.1	5261	1304	2.3	15.6
2002	2856	43005	15.1	5566	1395	2.4	16.2
2003	2890	42884	14.8	5738	1655	2.6	17.2
2004	2965	42836	14.4	5781	1478	2.4	16.9
2005	2989	43510	14.6	5347	1421	2.3	15.6
2006	3014	42708	14.2	4947	987	2.0	13.9
2007	3031	41259	13.6	4522	1509	2.0	14.6
2008	2977	37423	12.6	4438	1549	2.0	16.0
2009	2957	33883	11.5	4326	1189	1.9	16.3
2010	2967	32999	11.1	3553	1204	1.6	14.4
2011	2946	32479	11.0	3450	1084	1.5	14.0
2012	2969	33561	11.3	3666	735	1.5	13.1

^a^VMT data is annual and could not be separated into VMT traveled in adverse and non-adverse weather

**Fig. 1:**
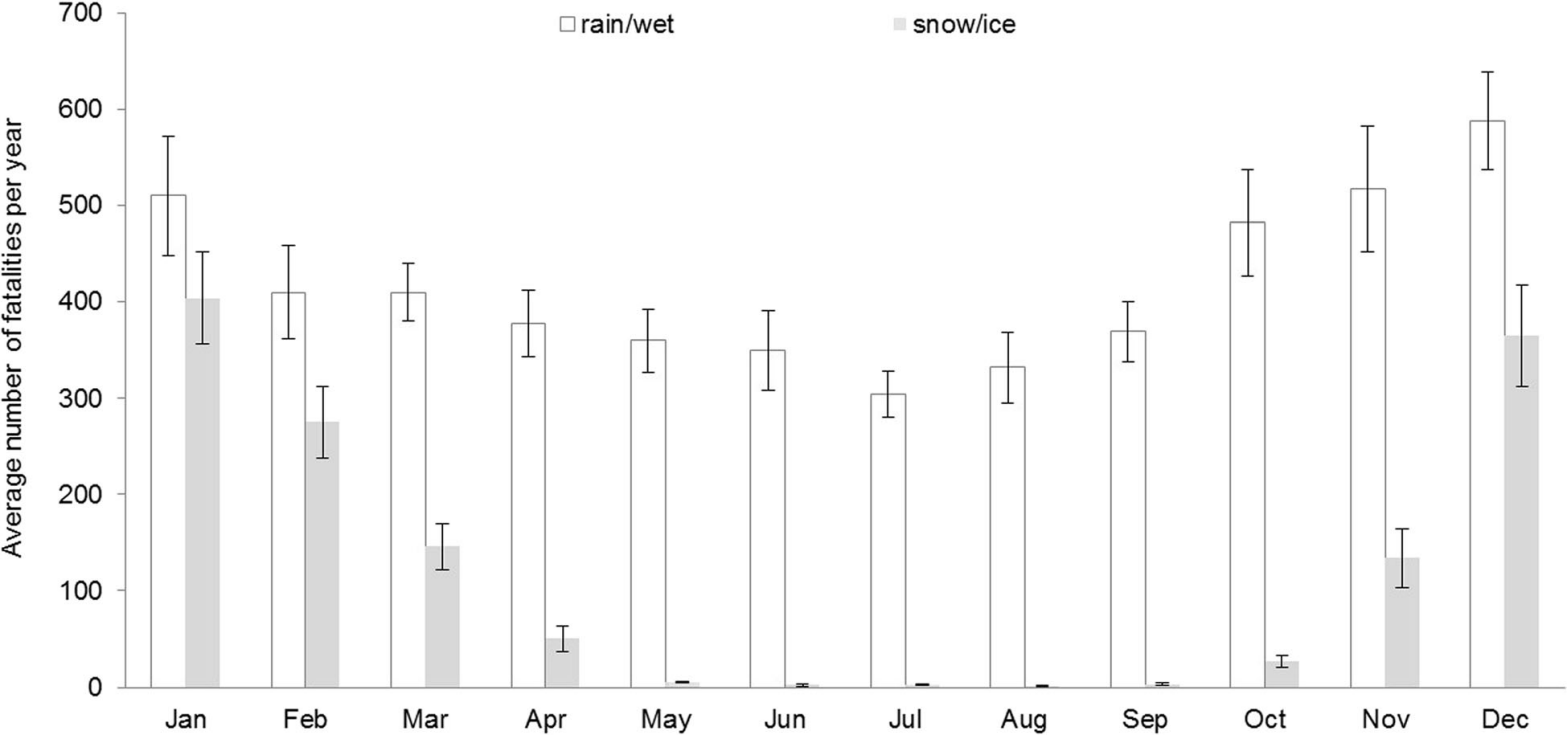
Average number of fatalities associated with adverse weather conditions by month (FARS, 1994-2012)

For the percentage of fatalities across age groups each year, we did not observe statistically significant differences between fatalities associated with adverse weather conditions and those that were not. We observed that rain/wet road conditions were reported across all months, compared to snow/icy road conditions that were predominantly reported during October to April (Fig. 1). On average during 1994–2012, the highest count of fatalities was observed in December (953.3, 95 % CI: 884, 1022) while the lowest was in July (305.3, 95 % CI: 309, 393). On average, 65 % of fatalities associated with adverse weather happened between November and April.

The odds ratios indicate if the known risk factors for fatal crashes were more likely to be reported with adverse weather conditions compared to fatalities when no adverse weather was reported (Table 2). Involvement of alcohol (OR = 0.83, 95 % CI: 0.82, 0.84) or drugs (OR = 0.74, 95 % CI: 0.71, 0.78), no use of restraint (OR = 0.93, 95 % CI: 0.92, 0.94), speeding (OR = 0.96, 95 % CI: 0.93, 0.98) and driving on urban roads (OR = 0.84, 95 % CI: 0.83, 0.86) were less likely to be reported for fatal crashes when adverse weather conditions were reported. Male drivers had a higher odds ratio (1.16, 95 % CI: 1.14, 1.17) of fatality associated with adverse weather. The odds ratio for poor driving record was 1.01 (95 % CI: 1.00, 1.02), with slight variation between the rain/wet and snow/icy conditions. The odds ratios for a fatal crash happening on a highway (OR = 1.10, 95 % CI: 1.09, 1.11) and in poor light conditions (OR = 1.11, 95 % CI: 1.09, 1.13) were higher under adverse weather conditions compared to non-adverse weather conditions.

**Table 2:** Odds ratios^a^ for risk factors commonly associated with fatal crashes occurring with adverse weather conditions

	Male driver	Alcohol involvement	Drug involvement	No restraint use	Speeding reported	Urban road	Poor driving record	Highway	Poor light condition
All adverse conditions	1.16	0.83	0.74	0.93	0.96	0.84	1.01	1.10	1.11
	(1.15, 1.17)	(0.82, 0.84)	(0.71, 0.78)	(0.92, 0.94)	(0.93, 0.98)	(0.83, 0.86)	(1.00, 1.02)	(1.09, 1.10)	(1.09, 1.13)
Rain or wet conditions	1.11	0.90	0.87	0.95	0.97	0.99	1.02	1.07	1.08
	(1.09, 1.13)	(0.89, 0.91)	(0.84, 0.90)	(0.94, 0.96)	(0.95, 0.99)	(0.97, 1.00)	(1.01, 1.03)	(1.07, 1.08)	(1.06, 1.10)
Snow or icy conditions	1.20	0.57	0.42	0.85	0.89	0.45	0.97	1.18	1.19
	(1.18, 1.21)	(0.55, 0.59)	(0.37, 0.47)	(0.83, 0.87)	(0.82, 0.96)	(0.44, 0.47)	(0.96, 0.99)	(1.17, 1.19)	(1.17, 1.22)

^a^Each odds ratio is the odds of the risk factor being reported in a crash which happened during adverse weather conditions divided by the odds of the risk factor occurring when no adverse weather conditions are reported

**Fig. 2:**
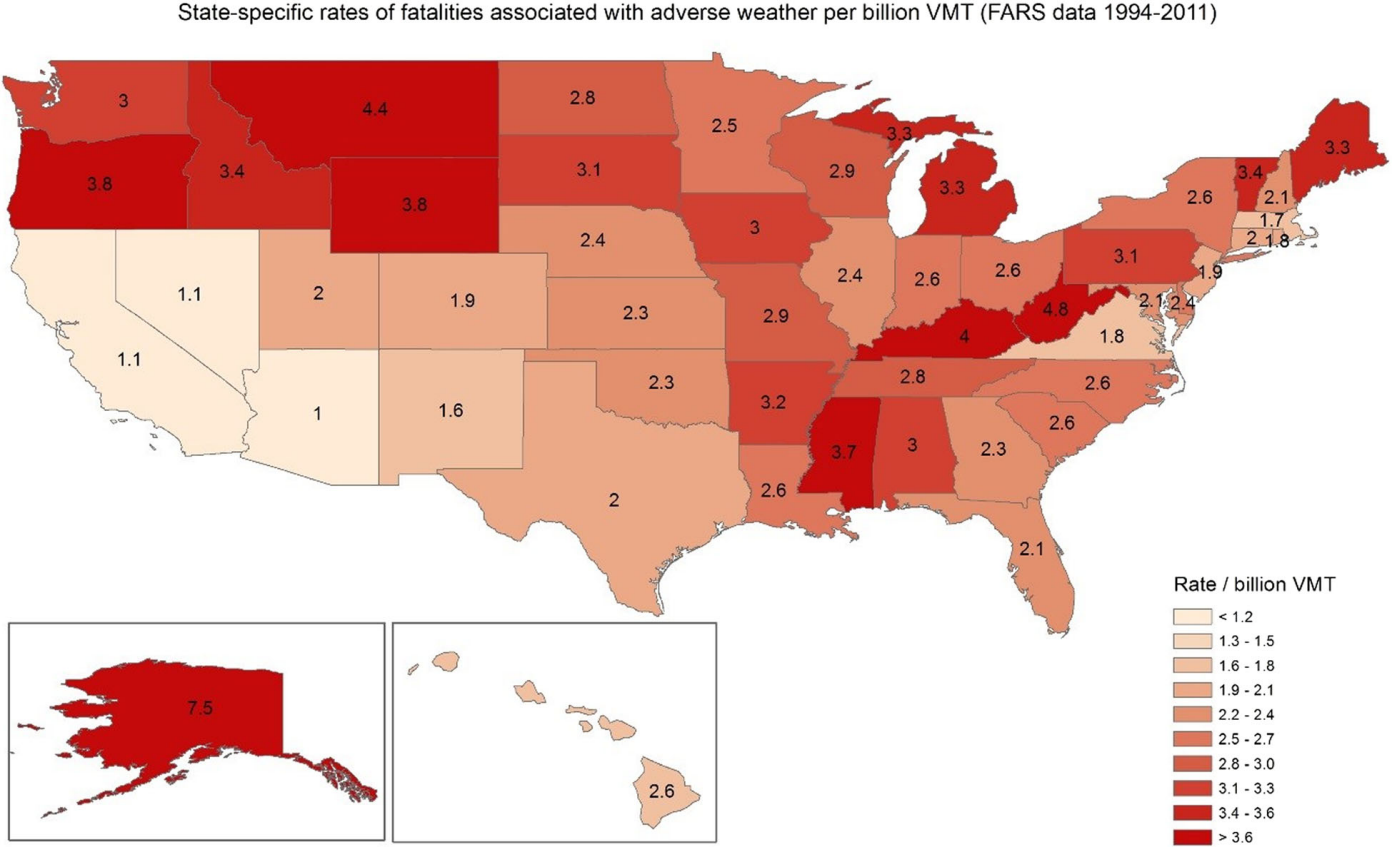
State-specific rates of fatal crashes associated with adverse weather conditions per billion Vehicle Miles Traveled, (FARS, 1994-2011)

**Fig. 3:**
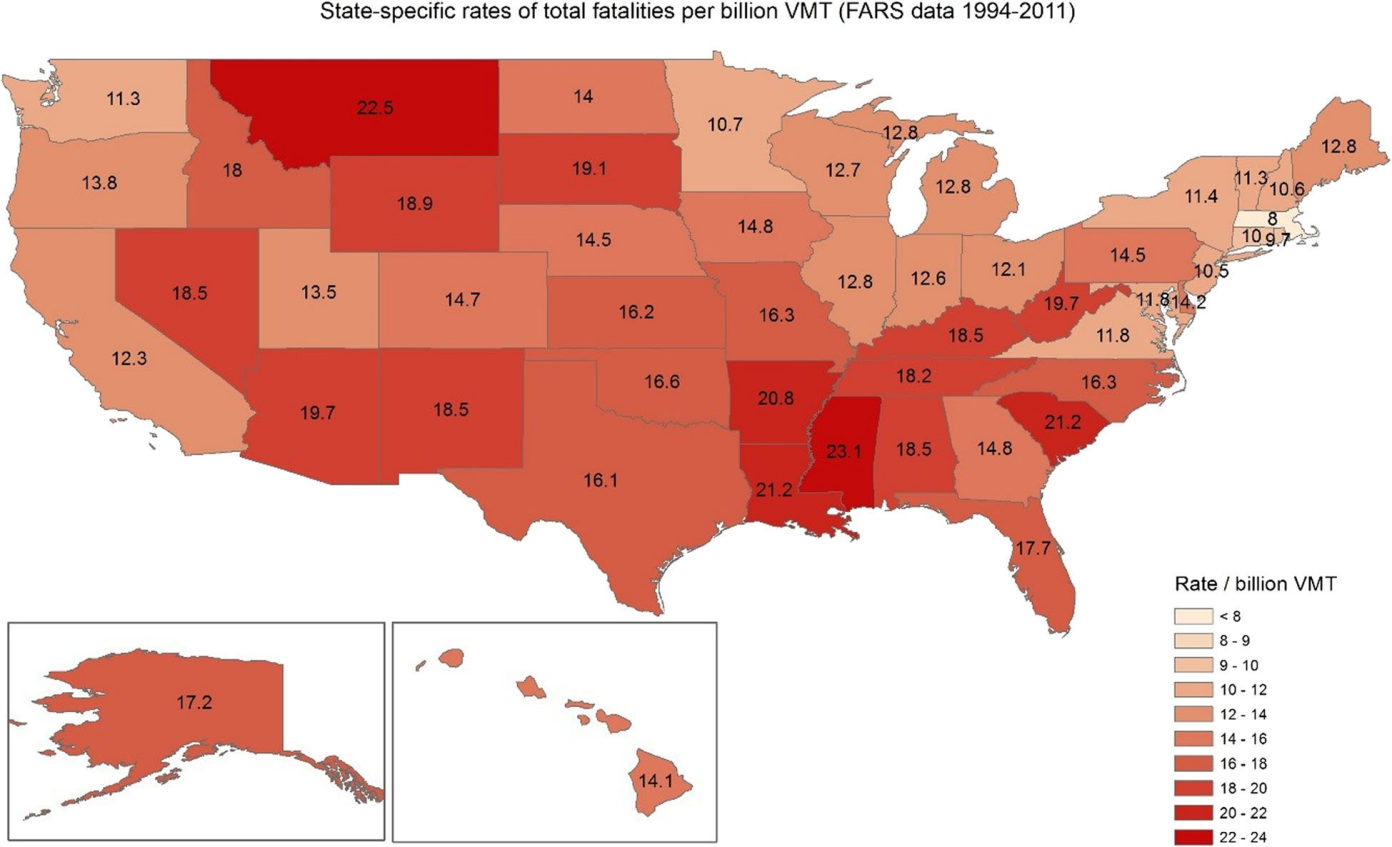
State-specific rates of fatal crashes per billion Vehicle Miles Traveled (FARS, 1994-2011)

Rates for all motor vehicle fatality were higher than rates of fatalities associated with adverse weather in the Southwest; opposite patterns were observed in the Pacific Northwest and some states in the Northeast (Figs. 2 and 3). The Spearman rank correlation between the two rates was 0.35 (*p* value = 0.01) indicating that the spatial pattern in fatality rates associated with adverse weather is different than the total fatality rates.

## Discussion

Using a census of fatal motor vehicle crashes in the United States from 1994 to 2012, we found a declining trend in total fatalities and fatalities associated with adverse weather, including its effects on road surfaces. Fatalities associated with adverse weather conditions averaged about 16 % of the total. Weather-related fatalities were found to vary both seasonally and geographically. We also found co-variation of adverse weather with some known risk factors.

The magnitude of fatal crashes associated with adverse weather was relatively higher in winter (October to March) compared to summer (April to September). This is plausible since nearly 70 % of the population reside and 74 % of the nation’s roads are located in snowy regions [11]. This could be attributed to precipitation in freezing conditions and fewer daylight hours in the winter months. This reasoning is strengthened by our findings that poor light conditions were more likely to be reported for adverse weather fatalities and the observed higher rates of fatalities associated with adverse weather among states in the Northern latitudes with shorter days. Some studies found snowfall to have a greater effect than rain in increasing both crash and injury rates [14, 20], although others indicate no significant difference between rainfall and snowfall in terms of injury collision or injury risk [13]. In adverse weather many more fatalities may occur at slower speeds than would otherwise be expected. One study in the Northeast US found that driving during winter months was a significant risk factor for crashes on slippery roads [21]. Under certain specific conditions (such as severe winter storms), the rate of localized motor vehicle crashes were found to increase substantially as compared to dry weather [22]. Overall the literature suggest a higher risk for injury crashes is associated with snowy and wintry conditions due to likely changes in road surface conditions, but this risk may be modified by region, and the impact on fatalities is unclear.

While geographic variation in overall motor vehicle fatality rates have been observed across the US since 1980 [23], we are unaware of any previous study that has examined the state-by-state occurrence of fatal weather-related crashes in the US. Clusters of weather-related crashes have been found in counties in Wisconsin that received more snow or rain [24]. We found that total fatal crashes and crash rate (per billion VMT) varied from state to state, and spatial distribution of adverse weather fatalities was different than the distribution of fatalities overall. This could be due to a number of factors. For example, the desert Southwest receives little rainfall or snow, so results indicating that Arizona has the lowest rate of fatal weather-related crashes is not surprising. Conversely, Alaska, which has long periods in which roads may be icy, had the highest fatality rate associated with adverse weather. We found that odds of fatal crashes on rural roads were higher when adverse weather was reported (compared to no adverse conditions), but we were unable to assess how potentially poorer access to health care or greater response time post crashes in rural areas would affect the fatality rates.

Another potentially important aspect of our findings is the distinction of adverse weather as an independent risk separate from other known risk factors [25, 26]. Known risk factors in fatal crashes that could interact with the injury host, the agent, or the driving environment include alcohol use [27], drug use [28], poor driving record [29], no restraint use [30], high speed [31], and poor light conditions [32–34]. In our analysis, alcohol or drug use, poor driving record, no restraint use, and speeding were less likely to co-occur in fatalities during adverse weather conditions suggesting the importance of adverse weather as an independent risk factor. It also supports the theory of risk compensation, which suggests that people typically adjust their behavior in response to their perceived level of risk (for example, driving more safely and slowly when environmental hazards are greater, and driving less carefully when people feel more protected, such as when they wear a seat belt) [35]. Our analysis also showed that adverse weather fatalities happened more often on highways, where speeds are higher, in poor light conditions, and among males. This concurs with existing evidence suggesting that reduced travel speeds are associated with lower fatality rates [36]; of increased crash risk when poor light conditions combine with adverse weather [20]; that males are at higher risk [12, 37]. These findings highlight the importance of further investigating any behavioral modifications that drivers tend to make while driving in adverse weather that influences the risk of fatal crashes. The use of observed meteorological data need to be used in future analysis to assess how intensity and frequency of precipitation may affect driving behavior.

This study has several limitations. The FARS database only includes fatal crashes, and no information on non-fatal crashes, which prevents us from assessing how adverse weather changes the likelihood of non-fatal crashes. We only had access to the subjective law enforcement assessment of local weather and road conditions, alcohol and drug involvement, speeding and seat belt use. Linking observed meteorological conditions at the time and location of fatal crashes should provide better characterization of adverse weather. We had access to annual VMT estimates only, and thus could only estimate annual fatality rates for all adverse weather condition. Driving patterns however change across seasons and fatality rates specific to VMT in winter and summer months could be important supplementary information. Estimation of fatality rates to specific weather type could be useful given the seasonal variability in adverse weather. The information on whether alcohol or drugs were involved in each crash was based on a combination of blood alcohol level tests and the subjective assessment of law enforcement personnel at the scene of crash, limiting our ability to ascertain between high and low levels of intoxication.

These findings have potential implication for research on how motor vehicle crashes related to adverse weather may be affected by climate change. Total annual and seasonal precipitation has already shifted in some areas of the US, and climate models predict this trend will continue [38] and incidence of severe weather to increase [39, 40]. Determining the impact of recent climate change on road traffic injuries is difficult, given lack of precise exposure information, though the number of deaths in vehicles from flash floods in Texas exhibited a general upward trend from 1959 to 2009 [41]. A study in Vancouver estimated an increase in collision counts by the mid-2050s due to greater rainfall intensity [42]. Downscaled climate models and local traffic models will need to be combined at appropriate spatial scales to determine localized impacts. While the potential impacts of climate change on fatal motor vehicle crashes is unclear, public health practitioners should be aware of the possibility of increased risk with changes in weather, especially those that occur suddenly and unexpectedly.

Another implication of the findings is that prevention of road traffic injuries related to adverse weather conditions may require specific prevention strategies. Returning to Haddon’s matrix can help lend insight into possible intervention strategies. In Haddon’s pioneering analysis, environmental conditions were named as factors affecting risk in each phase of the event. Another approach, aimed at highlighting the role of adverse weather in fatal crashes, could be to develop a matrix focused on factors specific to crashes in adverse weather. In Table 3 we present a modified matrix illustrating the ways in which adverse weather conditions might affect several factors relevant to road traffic injury prevention and control. The hosts in the matrix are people injured by road traffic crashes in which adverse weather plays a role; the agent is kinetic energy transferred from vehicles and objects in the environment to the host; the social environment includes relevant cultural and policy factors; and the physical environment includes physical factors in the vicinity of the crash. This matrix is meant to be neither prescriptive nor exhaustive but instead to frame a potential line of inquiry; potential interventions would need to be developed and vetted for efficacy.

**Table 3:** Conceptual Haddon matrix of factors in road traffic fatalities in the setting of adverse weather and road conditions

Phase	Host	Agent	Social environment	Physical environment
Pre-Event	Severe weather driving experience and training	Specific adverse weather equipment affecting performance and thus energy transfer (e.g., tire chains)	Transportation infrastructure (e.g., road network) preparedness for adverse weather. Adverse-weather speed limits	Weather-resilient signage. Road surface preparation (e.g., salt). Road and bridge design (e.g., drainage capacity)
Event	Ability to perform emergency and evasive maneuvers	Stopping distance. Anti-lock brakes. Vehicle performance characteristics. Safe airbag deployment		Collision barriers
Post-Event	Capacity to withstand adverse environmental and injury exposures	Vehicle crash notification system	Transportation planning and traffic flow management to facilitate emergency medical and safety response	Emergency medical service arrival and transport time. Feasibility of air transport

Some possible phase-specific factors affected by adverse weather and road conditions. Factors wherein adverse weather conditions are not considered a significant part of the causal pathway, e.g., use of active and passive restraints, are not listed

## Conclusion

While motor vehicle fatalities declined from 1994 to 2012, an adverse weather condition was reported at the time of crash for an annual average of 16 % of all fatalities. Among these fatalities associated with adverse weather conditions, the odds of some risk factors (e.g., poor light conditions, driving on highways) also being reported were high, while the odds of other risk factors (like alcohol and drug involvement, speeding, lack of restraint use, poor driving record) were low. These fatalities associated with adverse weather vary by season and different parts of the US. These findings have implications for future research on the potential risk of motor vehicle fatalities, either from impacts of adverse weather on driver behavior or from the events’ effects on pavement and the safety of roads.

## Additional file

Additional file 1: Online Supplement. (DOCX 17 kb)

## Abbreviations

CDC: Centers for Disease Control and Prevention
FHA: Fatality Analysis Reporting System
NHTSA: National Highway Traffic Safety Administration
