# Supplementary material for: Adverse weather conditions and fatal motor vehicle crashes in the United States, 1994-2012
Source: Environ Health. 2016 Nov 8;15:104. doi: 10.1186/s12940-016-0189-x (PMC5100176; doi:10.1186/s12940-016-0189-x)
Supplement: Additional file 1: — Online Supplement. (DOCX 17 kb) [file 12940_2016_189_MOESM1_ESM.docx]

**Online Supplement**

*Identification of adverse weather conditions*

From 1994 to 2006, the prevailing atmospheric condition that existed at the time of the crash was recorded in the variable WEATHER. The conditions that we considered as adverse were rain, sleet, snow, rain and fog, sleet, and fog. The conditions that we considered as non-adverse were clear, cloudy or no adverse atmospheric condition. From 2007 to 2009, the conditions snow and blowing snow were added to the WEATHER variable. These were included in our definition of adverse weather. From 2010 to 2011, two atmospheric conditions (primary and secondary) could be recorded on the crash report form at the time of the crash. We considered both WEATHER1 and WEATHER2 for these years. The weather elements included in the data were clear, cloudy, rain, sleet, hail, snow, and blowing snow. The weather elements not included were crashes during no additional atmospheric condition, fog, smog, smoke, severe crosswinds, blowing sand, soil, dirt, other, and not reported.

*Identification of adverse road conditions*

From 1994 to 2009, the roadway surface condition at the time of the crash was recorded in the variable SUR_COND. The roadway surface conditions that we considered as adverse were wet, snow or slush, and ice. The condition that we considered as non-adverse was dry road condition. The variable name changed to VSURCOND from 2010 onwards.

*Related factors*

There are other factors besides weather and road surface conditions that can affect fatalities during automobile crashes. Some important risk factors that have been associated with fatal motor vehicle crashes are: alcohol involvement, drug involvement, poor driving record, speeding, no restraint use, urban road, highway and poor light conditions. Information on these risk factors were obtained from each crash record in FARS, and were cross-tabulated with weather conditions to assess how these risk factors co-occur with adverse weather conditions.

**Alcohol involvement**: This was a binary indicator equal to one based on records whether the driver was drinking and is derived from data elements in the Vehicle and Person data files. If positive blood alcohol concentration was found or police-reported alcohol involvement, then a driver was classified as drinking. (Variables used were DR_DRINK, DRINKING).

**Drugs involvement**: This was a binary indicator equal to one based on records whether drugs were involved based on the judgment of law enforcement. (Variable used was DRUGS).

**Poor driving record**: This was a binary indicator equal to one if any of these conditions were met - (i) any previous crashes on record, (ii) any previous license suspensions or revocations, (iii) any previous DWI convictions, (iv) any previous speeding convictions, (v) any other previous moving violations or convictions for this driver that occurred within three years of the crash date. (Variables used were PREV_ACC, PREV_SUS, PREV_DWI, PREV_SPD, PREV_OTH).

**Speeding**: This was a binary indicator equal to one based on whether the speed the vehicle was traveling prior to the occurrence of the crash was above the posted speed limit at the location of crash as reported by the investigating officer. (Variables used were VSPD_LIM, TRAV_SPD).

**No restraint use**: This was a binary indicator equal to one if no restraint equipment were used by the occupant, or no helmet was used by a motorcyclist at the time of the crash. (Variable used was REST_USE).

**Urban road**: This was a binary indicator using the data element identifying the functional classification of the traffic way on which the crash occurred. If the road segment was indicated as an ‘urban’ code, then the indicator was assigned a value of one. (Variable used was ROAD_FNC).

**Highway**: This was a binary indicator equal to one based on the route signing (interstate, US route or state route) of the traffic way on which the crash occurred. (Variables used was ROUTE).

**Poor light conditions**: This was a binary indicator equal to one based on whether the type/level of light that existed at the time of the crash was insufficient (dark, dusk or dawn) or during daylight. (Variable used was LGT_COND).
